# Relieving total pain in an adolescent: a case report

**DOI:** 10.1186/s13104-018-3368-8

**Published:** 2018-05-02

**Authors:** Tharin Phenwan

**Affiliations:** Walailak University School of Medicine, Nakhon Si Thammarat, 80161 Thailand

**Keywords:** Palliative care, Adolescence, Total pain, Case report

## Abstract

**Background:**

Total pain is a concept that approaches pain holistically: physically, psychologically, socially, and spiritually. Any individual may experience pain in each domain at a different level. This is the case report of an adolescent who suffered from total pain and how his healthcare team and peers helped to relieve it.

**Case presentation:**

A 15-years-old Thai male was diagnosed with recurrent T-cell lymphoma and readmitted to hospital. He was admitted to an adult ward and suffered from pain due to his disease and from the fear of being alienated. As a result, he had an existential crisis. His parents felt unsure whether they or the patient should make the medical decisions and advance care plan.

**Conclusions:**

This case report emphasises the importance of total pain assessment in the relief of total pain in an adolescent whose needs are different from both children and adults. It also highlights the role of medical decision-making in adolescents and the importance of the social support of peers in the alleviation of pain.

## Background

**Fig. 1:**
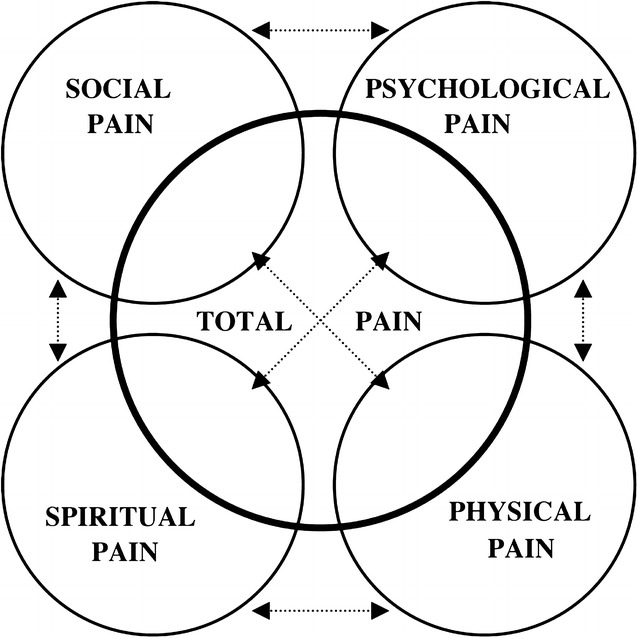
Total pain components (picture from [3])

Total pain is the concept of approaching pain from all aspects of life: physical, psychological, social, and spiritual [1–3] (Fig. 1) [4]. It is also one of the principles of palliative care, an approach that aims to improve quality of life of the patients and their family who are facing life-threatening illnesses. Any individual may experience pain in each domain at a different level, especially adolescents who will require extra care in the psychosocial aspect of pain [5–7]. This case report tells the story of an adolescent who suffered total pain and how his healthcare team and peers helped to relieve it.

## Case presentation

A 15-year-old Thai male who had recurrent T-cell lymphoma. He was diagnosed with the disease 3 years earlier, and treated with 6 cycles of chemotherapy. Regular follow-ups showed no signs of recurrence until 1 week before admission when he developed progressive dyspnoea. Chest X-ray at a provincial hospital showed massive right lung pleural effusion and an anterior mediastinal mass was detected. His GP referred him to a tertiary level hospital for further care. Upon admission, hospital regulations stated that a patient who is over 15 years of age must be admitted to an adult ward; hence he was transferred to an internal medicine ward. During his first night, he witnessed the death of a patient who had a cardiac arrest. The nightshift team performed CPR, intubation, and various other lifesaving procedures. The boy witnessed all of this, and was deeply traumatised. After which, he had a complete psychotic breakdown and did not stop screaming until his doctor sedated him. When he woke up in the night, he continued screaming; thus he needed to be sedated again, and nurses had to put a curtain around his bed, blocking him off from the rest of the ward. At the time of his palliative care team consultation, the patient was restless with a palliative performance scale (PPS) of 40, and had suicidal tendencies. He kept screaming whenever he woke up, calling constantly for his mother to end his misery and yelling, “*I don’t belong in this place*”. Further history taking revealed that he had every aspect of total pain: physical, emotional, social, and spiritual.

### Physical pain

His pain was dull and occurred mainly around the chest area, without any radiation. His baseline pain score was 9 out of 10 while the worst pain was 10 out of 10. He also complained of dyspnoea which worsened when he talked a lot or felt anxious.

### Psychological pain

A psychiatrist assessed him and diagnosed him with adjustment disorder from both his progressive illness and the traumatising experience of witnessing his first death.

### Social pain

The patient had a high-achiever profile. He was the top of his class, played basketball at the regional level, and was loved by everyone. He constantly talked about his fear of being “left out” of his group of friends because he could not attend school anymore, and that they were moving forward while he was “stuck here”. His mother is a palliative care nurse and was torn between her role as a nurse and the mother of a dying child. She felt unsure about the advance care plan (ACP), and whether she should make the decisions for her son or let him decide for himself. His father and sister also concurred.

### Spiritual pain

Spirituality is described as an individual’s search for meaning and purpose in life, the experience of the transcendent, and also one’s connectedness with others: his or herself, nature, and to the sacred realms, inside as well as outside of traditional religion [8]. In the patient’s case, his sources of strength were his family, academic interests, especially in English, and his friends. He had an existential crisis and felt that his life was meaningless because he was no longer who he had once been, “*I am alone in a world full of dying old men*”. He felt it would be better for his misery to end than to keep existing with no meaning. He constantly asked his mother when would he die and would he end up like that man he saw in the ward.

### Management

For his physical pain, we used the WHO analgesic ladder to slowly titrate his medications with morphine and other adjuvants. Along with solving his other pain components together, his pain and dyspnoea were well-controlled with subcutaneous morphine and fentanyl patch 3 days after the consultation. As for his psychological pain, to prevent him from further psychic trauma, we moved him to a private room in a paediatric ward, where the environment was more familiar to him. He seemed a lot more at ease there. We also prescribed lorazepam, haloperidol, and subcutaneous midazolam for his anxiety. For his social and spiritual pain, the team contacted his teachers and friends, all of whom were worried sick about him. A few days later, his teachers, classmates, and underclassmen paid him a visit. They made get-well cards for him and performed *Bai Sri Su Kwan*: a Thai blessing ceremony that celebrates important events or conveys wishes for an individual’s well-being. His friends brought him learning materials and homework and kept contact with him via social media so that he would not feel left out again. They also made it clear that he was still part of their group, as always. One nurse on our team also offered to teach him English, provided that he was not too exhausted.

For his ACP, after the family meeting, everyone in his family agreed that the patient was very mature for a boy his age and they agreed that it would be in his best interest to let him decide the goal of care by himself. After the full explanation of the prognosis, he chose to be cared at home, alongside his friends and family. The team then prepared the essential medications, made a comprehensive referral note, and contacted the district hospital beforehand so that when he arrived home there would be a community nurse regularly checking up on his symptoms and refilling his subcutaneous medications. He passed away comfortably a few days after he was discharged, in his bedroom, with his best friend and mother by his side.

## Discussion and conclusions

Total pain is a concept that was coined by Dame Cicely Saunders. It is composed of the physical, psychological, and social, with a spiritual component [2]. Any individual may experience pain in each domain with different severity [9]. Even though there is a gold standard for pain control, i.e., the WHO analgesic ladder, it focuses mainly on the physical aspect of pain, thus further evaluation of the other domains may be needed [7, 10]. Even though there are several tools for the psychological and spiritual pain, healthcare providers usually underutilise it thus making it harder to assess patients comprehensively [11]. This case report is an example of the manifestation of total pain in an adolescent, which is a transitional stage of life with complicated needs [12]. Studies show that young patients, especially adolescents, require different care than other age groups [5, 6, 12–14]. Since their illness usually disrupts their normal physical and psychological development, most of the time adolescents’ concerns are psychosocial: fear of social isolation from their peers [15], maintaining their self-image [16], and the attainment of independence [13, 14]. However, these needs, especially from the psychosocial aspect, are usually under met [7]. Their spirituality is frequently overlooked as well [10]. Key developments in comprehensive care in adolescents consist of understanding adolescents’ unique features and also flexibility of care [6]. Other keys are adequate symptom control [17] and thorough and honest discussions concerning medical-decision making [18]. As for the patients’ autonomy, while there are ongoing debates between balancing the teenager’s autonomy with their parents’, the most important consideration is rather that key members are involved in the discussion and that joint decision-making is undertaken [12, 13, 19]. Friends also have a huge impact on their care since one of the unique types of pain for adolescents with cancer is the need for normality: patients simply want to blend back in with friends, just like before their illness [5, 15]. Finally, according to The United Nations Convention on the Rights of the Child (CRC), a child is still considered a child until he/she reaches 18 years of age [20]. Thus, adolescents, even though they are different than children and adults, should not be treated in adult care as in this case. This case report emphasises the importance of total pain assessment in order to relieve the different aspects of pain in adolescents who require different care from other age groups. It also highlights the medical decision-making process in adolescents, flexibility of care, and the importance of social support from peers in helping adolescents get through their ordeals.

## Abbreviations

ACP: advance care plan
PPS: palliative performance scale
